# Reliability and validity of the Finnish version of the American Shoulder and Elbow Surgeons Standardized Shoulder Assessment Form, patient self-report section

**DOI:** 10.1186/1471-2474-15-272

**Published:** 2014-08-11

**Authors:** Kirsi Piitulainen, Juha Paloneva, Jari Ylinen, Hannu Kautiainen, Arja Häkkinen

**Affiliations:** Department of Health Sciences, University of Jyvaskyla, PL 35, 40014 Jyvaskyla, Finland; Department of Physical Medicine and Rehabilitation, Central Finland Health Care District, Jyvaskyla, Finland; Department of Surgery, Central Finland Health Care District, Jyvaskyla, Finland; Unit of Primary Health Care, Helsinki University Central Hospital, Helsinki, Finland; Department of General Practice, University of Helsinki, Helsinki, Finland; Unit of Primary Health Care, Kuopio University Hospital, Kuopio, Finland

**Keywords:** The American Shoulder and Elbow Surgeons Standardized Assessment Form (ASES), Shoulder pain, Reliability, Validity

## Abstract

**Background:**

The American Shoulder and Elbow Surgeons Standardized Shoulder Assessment Form (ASES) is one of the most widely used shoulder outcome tools in clinical work and in scientific studies. However, it has not been validated in the Finnish language. The aims of this study were to cross-culturally adapt the ASES to the Finnish language and to study the psychometric properties of the self-report section of the ASES.

**Methods:**

A total of 105 patients with shoulder symptoms answered the questionnaires of the ASES, a single disability question, the Simple Shoulder Test (SST), and the Short-Form 36 Health Survey (SF-36). The reliability of the ASES questionnaire was studied using a test-retest procedure at 2-week intervals. Psychometric assessment was performed by testing the construct validity, internal consistency, the criterion validity, and the convergent validity of the ASES.

**Results:**

The reproducibility and internal consistency of the ASES were 0.83 (95% CI 0.70 to 0.90) and 0.88 (95% Cl 0.84 to 0.91). There were no significant differences between the diagnostic groups in the pain scores from the ASES, and the function score was significantly higher in the instability group compared to the other groups. The convergent validity of the ASES correlated with the SST, *r* = 0.73 (p < 0.001); the single disability question, *r* = -0.74 (p < 0.001); and the Physical Component Score of the SF-36, *r* = 0.57 (p < 0.001).

**Conclusions:**

The Finnish version of the ASES proved to be a reliable and valid tool for assessing shoulder disabilities in patients with different shoulder diagnoses, including rotator cuff disease, instability, and osteoarthritis.

**Electronic supplementary material:**

The online version of this article (doi:10.1186/1471-2474-15-272) contains supplementary material, which is available to authorized users.

## Background

Shoulder pain is the third most common musculoskeletal problem after low back pain and neck pain [1]. Shoulder pain is responsible for a remarkable amount of sick leave in western countries [2]. One-third of the population over 30 years of age reported shoulder pain during the last month [3]. When treating these patients, it is crucial to obtain information from the patient’s point of view to assess the level of symptom severity and the level of disability.

There are two types of commonly used patient-based outcome tools. First, the generic measures (e.g., SF-36, EuroQol, and WHOQOL) evaluate general health, overall disability, and quality of life. However, they are not sensitive enough to react to clinically relevant changes in a specific disease [4]. Second, disease-specific measurement instruments connect the symptoms and disability to a specific disorder. One of the most frequently used questionnaires concerning the shoulder is the self-report section of the American Shoulder and Elbow Surgeons Standardized Shoulder Assessment Form (ASES) [5]. It has been validated in many languages and is considered to be a reliable, valid, and responsive outcome tool [5–12]. The psychometric properties of the ASES are reported to be acceptable for clinical use throughout every target language [6–8, 11, 12].

The ASES questionnaire has been used extensively in Finland. In addition it is easy and quick for a patient to complete. However, the ASES questionnaire has not been validated in the Finnish language. Compared to other questionnaires for the functional evaluation of the shoulder, e.g. the Disabilities of the Arm, Shoulder and Hand Questionnaire (DASH), which was developed to be used in patients with any disorder in any joint of the upper limbs, the ASES is more joint-specific instrument and therefore, more responsive and effective as a shoulder research tool [13]. The purpose of this study was to cross-culturally adapt the self-report section of the ASES questionnaire and to demonstrate the reliability and validity of the ASES among Finnish-speaking patients with shoulder pain.

## Methods

### Translation and cross-cultural adaptation

The translation and cross-cultural adaptation were performed based on the guidelines proposed by Beaton et al. [14]. The first stage was an independent translation (English to Finnish) of the self-report section of the ASES by two professionals (each with Finnish as their first language). In the second stage, synthesis of the two translations was performed. In the third stage, a person not working in the field of medicine, whose first language is English, and who masters the linguistic and cultural aspects of the Finnish language, back-translated (Finnish to English) the synthesised version blinded to the purpose of the instrument. In the fourth stage, the translation of the Finnish version of the ASES was accepted by an expert committee. The pre-final version of the ASES was tested in few subjects with shoulder problems to probe about the understanding of the questionnaire. As none of the comments required changes in this final stage of the adaptation, the equivalence of the Finnish questionnaire was ensured. Finally, the form was tested in a population of 128 patients with various shoulder disorders [15]. The Finnish version of the ASES is available on the Internet page of the Clinical Musculoskeletal Diseases Research Group of the Central Finland Health Care District (http://www.ksshp.fi/fi-FI/Ammattilaiselle/TULEStutkimus/Clinical_Musculoskeletal_Diseases_Resear(45030)) and in this article [see Additional file 1].

### Patients, setting, and data collection

The psychometric characteristics of the Finnish version of the patient self-report section of the ASES questionnaire were examined in a sample of 105 consecutive patients who were clinically diagnosed with a shoulder disorder and referred for specialised care (the outpatient clinics in the Department of Physical Medicine and Rehabilitation or the Department of Orthopaedics and Traumatology in Central Finland Hospital, Jyväskylä, Finland). Our aim was to recruit a sample of at least 100 patients. The shoulder diagnoses were classified on the basis of information retrieved from the patient’s medical records and, if needed, radiologic examinations (e.g., plain radiographs or magnetic resonance imaging) by an orthopaedic surgeon (JP). The inclusion criteria were age over 18 years, shoulder symptoms, and ability to communicate in the written Finnish language. The only exclusion criterion was previous surgery in the affected shoulder less than 1 year ago. The patients answered a questionnaire package that included the self-report section of the ASES, the Simple Shoulder Test (SST) [16], the Short-Form 36 Health Survey (SF-36) [17], and clinical and socio-demographic data. The self-report section of the ASES questionnaire was administered twice. The first questionnaires were mailed to the patients and the patients completed those 2 weeks before arriving at the outpatient clinic of Physical and Rehabilitation Medicine or orthopaedic surgery and again a second time when they came to the clinic. At the clinic the patients were contacted personally by a physiotherapist and asked to complete the ASES questionnaire for the second time.

### Measurements

The self-report section of the ASES form is divided into two sections: pain and activities of daily living. The total ASES score is derived from a pain question using the Visual Analogue Scale (VAS) ranging from 0 mm (no pain) to 100 mm (worst pain), in addition to function during activities of daily living (1. Put on a coat, 2. Sleep on your painful shoulder, 3. Wash back, 4. Manage toileting, 5. Comb hair, 6. Reach a high shelf, 7. Lift 10 lb above shoulder, 8. Throw a ball overhand, 9. Do usual work, and 10. Do usual sport). These activities of daily living were assessed for each shoulder separately, and the 10 items were graded on a 4-point ordinal (Likert) scale. Scores ranged from 0 (unable to do the activity) to 3 (no difficulty in performing the activity). The pain score and the cumulative activities of daily living (ADL) score were weighted equally (50 points each) and combined for a total score (possible 100 points). The ASES score is equal to 5 ([100 - ASES pain VAS]/10 + ASES Cumulative ADL score/3). A single disability question (“How severe was your shoulder disability during the last week?”), the shoulder-specific Simple Shoulder Test (SST) [16], and the generic Short-Form 36 Health Survey (SF-36) [17] were used to check the convergent validity. The aforementioned SST has not been validated in the Finnish language; unlike the SF-36 has been validated [18]. The patients completed the ten items of activities of daily living in relation to both shoulders to find out how many patients had disorders in both shoulders, though these results are not reported in the present study. A few patients had both shoulders affected, but in the analysis we chose the shoulder for which the patient had visited the outpatient clinic. The patients also answered an additional question about whether their shoulder symptoms had been stable, improved, or worsened during the past 2 weeks. According to these answers, the patients were divided into three groups.

The patients were divided into four categories according to the clinical diagnosis made in the outpatient clinics: rotator cuff disease, osteoarthritis of the glenohumeral or acromioclavicular joint, instability, and other.

### Statistics

The results are expressed as means with standard deviation (SD) or with 95% confidence intervals (95% CIs), as counts with percentages, or frequency distributions. The 95% CIs were obtained by bias-corrected bootstrapping (5000 replications). The “floor value” was defined as the worst possible value of the item or as the minimum total value of the scale. The “ceiling value” was the best possible value of the item or the maximum total value of the scale. The reliability of the scales was evaluated by calculating the intra-class correlation coefficient (ICC) and coefficient of reproducibility with the bias corrected and accelerated bootstrapping (5000 replications) confidence intervals. The internal consistency was estimated by calculating Cronbach’s alpha. Item analysis of the ASES scales was performed by analysing the item discriminating power (corrected item correlation) and the item difficulty (item mean) depicted by the explanatory data analysis. Factor structure among the ASES items was analysed using a factor analysis with varimax rotation. Effect size (“d”) was calculated by using the method for paired samples: mean baseline scores minus mean follow-up scores, divided by the pooled standard deviation. Effect size of 0.20 was considered small, 0.50 medium and 0.80 large. 95 percent confidence intervals (95% CI) were obtained by bias-corrected bootstrapping (5000 replications). The correlation coefficients between the ASES and other patient-reported outcomes were calculated by the Spearman method using Sidak-adjusted probabilities.

### Ethics

The study was approved by the ethics board of the Central Finland Health Care District (November 23, 2005, Dnro 46/2005). Written informed consent was obtained from all participants.

## Results

A total of 105 patients were enrolled in the study (mean age 52 years, range 18-88). The mean (SD) shoulder pain was 56 (28) mm. The most common reason for shoulder pain was rotator cuff disease (41%). The demographic and clinical data of the study group are shown in Table 1.

**Table 1:** **Socio-demographic and clinical data of patients with shoulder disorders**

Variables	Values (N = 105)
Males, n (%)	60 (57)
Age, years, mean (SD)	52 (18)
Body mass index, mean (SD)	28 (5)
Education, years, mean (SD)	13 (4)
Employed, n (%)	38 (36)
Symptomatic shoulder, n (%)	
Right	65 (62)
Left	40 (38)
Pain, VAS (0-100), mean (SD)	
Shoulder	56 (28)
Upper limb	25 (33)
Neck	19 (26)
Back	16 (26)
Duration of shoulder pain, months, mean (SD)	56 (79)
Shoulder trauma, n (%)	51 (49)
Diagnosis, n (%)	
Rotator cuff disease	43 (41)
Glenohumeral or acromioclavicular arthritis	27 (26)
Glenohumeral instability	23 (22)
Other	12 (11)

*SD* standard deviation, *VAS* visual analogue scale.

Table 2 shows the floor and ceiling values of the initial assessment. The floor value was reached by five patients in the pain score of the ASES but not in the function score or in the total ASES index. Three patients reached the ceiling value in the pain section and one patient in the function score but not in the total ASES index. The total ASES score ranged from 2 to 99.

**Table 2:** **Reproducibility of the ASES index**

	Baseline				Change from first to second measurement	Reproducibility	
	Mean (SD)	Range	Floor* N (%)	Ceiling** N (%)	Mean (95% CI) [Effect Size]	ICC (95% CI)	CR (95% CI)
Pain score, all patients (N = 105)	21.9 (14.1)	0-50	5(5)	3(3)	4.0 (1.8 to 6.1) [0.26]	0.66 (0.52 to 0.77)	23 (19 to 28)
Improved (N = 25)	26.9 (14.4)	0-48	2(8)	0(0)	5.8 (0.5 to 11.2) [0.42]	0.50 (0.04 to 0.77)	27 (19 to 38)
Stable (N = 55)	22.3 (14.7)	0-50	2(4)	3(5)	3.9 (0.9 to 7.0) [0.27]	0.68 (0.46 to 0.82)	23 (17 to 29)
Worsened (N = 25)	15.9 (9.8)	0-36	1(4)	0(0)	2.2 (-1.6 to 6.0) [0.19]	0.67 (0.37 to 0.83)	18 (14 to 23)
Function score, all patients (N = 105)	25.5 (11.5)	2-50	0(0)	1(1)	0.2 (-1.3 to 1.6) [0.01]	0.81 (0.71 to 0.88)	14 (11 to 18)
Improved (N = 25)	27.5 (11.4)	3-46	0(0)	0(0)	2.6 (-0.1 to 5.3)[0.23]	0.81 (0.51 to 0.93)	13 (8 to 20)
Stable (N = 55)	26.0 (11.3)	3-50	0(0)	1(2)	-1.5 (-3.4 to 0.4) [0.13]	0.83 (0.64 to 0.92)	14 (10 to 19)
Worsened (N = 25)	22.5 (12.1)	1-40	0(0)	0(0)	1.5 (-1.9 to 4.9) [0.12]	0.79 (0.57 to 0.90)	16 (11 to 22)
Total ASES, all patients (N = 105)	47.4 (22.8)	2-99	0(0)	0(0)	4.1 (1.4 to 6.9) [0.18]	0.79 (0.69 to 0.86)	29 (25 to 35)
Improved (N = 25)	54.5 (24.1)	3-93	0(0)	0(0)	8.5 (1.5 to 13.4 )[0.37]	0.69 (0.27 to 0.87)	36 (24 to 52)
Stable (N = 55)	48.3 (22.8)	7-99	0(0)	0(0)	2.4 (-1.2 to 5.9) [0.10]	0.83 (0.70 to 0.90)	26 (21 to 31)
Worsened (N = 25)	38.5 (19.2)	2-73	0(0)	0(0)	3.7 (-2.1 to 9.4) [0.17]	0.77 (0.59 to 0.87)	28 (21 to 35)

*Worst possible value (Pain and function: 0, Total ASES: 0) of the item or minimum total value of the scale.

**Best possible value (Pain and function: 50, Total ASES: 100) of the item or maximum total value of the scale.

*ASES* American Shoulder and Elbow Surgeons Standardized Shoulder Assessment Form, *ICC* intra-class correlation coefficient, *CR* coefficient of repeatability.

When the questionnaire was administered for the first time, the mean (SD) total ASES score was 48 (23) for the patients with shoulder symptoms that had been stable between the first and the second measurement. For these patients, the reproducibility intra-class correlation coefficient was 0.83 (95% Cl = 0.70 to 0.90). For the patients with shoulder symptoms that had improved, the reproducibility ICC was 0.69 (0.27 to 0.87). For the patients with worsened symptoms, the reproducibility ICC was 0.77 (0.59 to 0.87) (Table 2).

**Figure 1:**
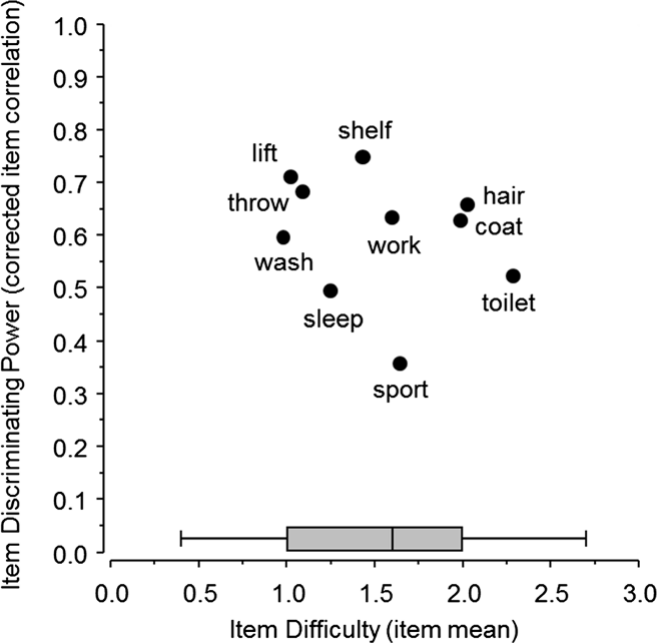
**Item analysis for the function items of the ASES. The bar denotes the median and interquartile range.**

The internal consistency estimate of Cronbach’s alpha was 0.88 (95% Cl 0.84 to 0.91). The item analysis of the ASES showed that item 6 (reaching a high shelf) had the highest corrected item correlation, whereas item 10 (doing usual sport) had the lowest corrected item correlation. In addition, item 3 (washing back) had the lowest item means, and item 4 (managing toileting) had the highest item means (Figure 1).

The factor analysis performed for construct validity showed that ASES was loaded on one factor that explained 66% of the total variance.

**Figure 2:**
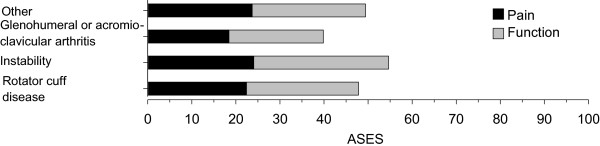
**Pain and function scores of the ASES index in different diagnosis groups.**

The total ASES index was the lowest in the glenohumeral or acromioclavicular arthritis group and the highest in the instability group. There was no statistical difference between the diagnostic groups in pain score, and the function score was significantly higher in the instability group compared to the other groups (p = 0.035) (Figure 2).

The baseline data are presented in Table 3. The correlations between the total ASES index and the SST scale and the single disability question (How severe was your shoulder disability during the last week) were 0.73 (p < 0.001) and -0.74 (p < 0.001). The mean shoulder disability scored by a single disability question was 54 (28). The correlations between the total ASES index and Physical Functioning, Role Physical, Role Emotional, Social Functioning and Bodily Pain from the SF-36 were statistically significant (Table 3). When the eight dimensions of the SF-36 were aggregated into summary scores, the correlations between the total ASES score and the Physical Component Summary and Mental Component Summary of the SF-36 were 0.57 (p < 0.001) and 0.21 (p = ns).

**Table 3:** **Disability and health-related quality of life and their correlations with the patient self-report section of the ASES**

	Mean (SD)	Correlations		
		**The total ASES**	**Pain score**	**Function score**
**SST (scale 0-12)**	5 (4)	0.73***	0.54***	0.81***
**A single disability question (scale 0-100)**	54 (28)	– 0.74***	– 0.67***	– 0.68***
**Dimensions of SF-36 (scale 0-100)**				
Physical Functioning	64 (25)	0.51***	0.38**	0.57***
General Health	58 (22)	0.27	0.22	0.32*
Vitality	60 (21)	0.58	0.21	0.32*
Mental Health	73 (21)	0.26	0.23	0.27
Role Physical	36 (39)	0.49***	0.41***	0.47***
Role Emotional	67 (42)	0.37**	0.28	0.42***
Social Functioning	75 (26)	0.44***	0.37**	0.46***
Bodily Pain	41 (21)	0.68***	0.630***	0.58***
**Summary Score of SF-36 (scale 0-100)**				
PCS	36 (10)	0.57***	0.48***	0.56***
MCS	52 (12)	0.21	0.17	0.25

*p < 0.05, **p < 0.01 and ***p < 0.001. Sidak adjusted probability.

*SD* standard deviation, *ASES* American Shoulder and Elbow Surgeons Standardized Shoulder Assessment Form, *SST* Simple Shoulder Test, *SF-36* Short Form 36 Health Survey, *PCS* Physical Component Score, *MCS* Mental Component Score.

During the translation process from English to Finnish and backward translation into English only minor linguistic and cultural differences between the translations emerged. The question of activities of daily living about lifting 10 lbs above the shoulder was adapted to the metric system. The original ASES uses the U.S. Unit system. The translated weight is 4 kg in our study.

## Discussion

In the present study, we assessed the cross-cultural adaptation and the psychometric properties of the self-report section of the ASES questionnaire to the Finnish language. We demonstrated that this version of the ASES has good reliability and validity.

It has been suggested that a questionnaire reaching a floor or ceiling value of over 15% should be omitted [14]. The present study had even lower floor and ceiling effects than 15%. One possible interpretation of this might be that a real floor or ceiling effect does not exist when using the Finnish ASES questionnaire. Kocher et al. [9] examined the floor and ceiling effect of the total ASES scale with different patient subsets (shoulder instability, rotator cuff disease, glenohumeral arthritis), and they found that only 1.3% of the patients with shoulder instability had a ceiling effect. Thus, the ASES score seems to have enough categories to discriminate the patients with different disability levels and changes.

In the present study, the baseline values in the stable, improved, and worsened groups were consistent. By dividing the patients into three groups it was possible to find out, if the ASES could detect differences between patients who have reported to be stable and those whose symptoms have been changed. The change was statistically significant only in the improved group (Table 2). The reproducibility ICC of the total ASES index in all patients was 0.79 (95% Cl: 0.69 to 0.86), but it varied between moderate and good in the three groups. The reproducibility ICC (95% Cl) was 0.83 (0.70 to 0.90) in the stable group (Table 2). According to Portney and Watkins [19], an ICC > 0.75 indicates an acceptable test-retest reliability score. Although the time interval between the first and the second measurement varies from 1 day to 4 weeks, the reproducibility ICC is ≥0.84 in the previous studies (Table 4). This indicates that test-retest reliability of the ASES is quite high and stable in all studied languages [6, 7, 9–12].

**Table 4:** **Summary of translation, cultural adaptation and validation studies of the ASES**

	Number of subjects (age range, years)	Language/validation study	Time interval between the first and the second measurement	Reproducibility ICC (95% Cl)	Internal consistency (Cronbach’s alpha)	Convergent validity ASES and other questionnaire	Convergent validity ASES and SF-36 PCS	Convergent validity ASES and SF-36 MCS
Piitulainen K et al. present data	n = 105	Finnish	2 weeks	0.83 (0.70 to 0.90), n = 55	0.88 (0.84 to 0.91)	SST		
						*r* = 0.73	*r* = 0.57	*r* = 0.21
	(18-88)							
				0.79 (0.69 to 0.86), n = 105		p < 0.001	p < 0.001	ns
Celik D et al. [6]	n = 63	Turkish	3-7 days	0.94	0.88	SPADI		
	(18–74)							
						*r* = – 0.82	*r* = 0.02	*r* = 0.53
						p < 0.001	p = 0.82	p < 0.000
Yahia A et al. [12]	n = 80	Arabic	1-3 days n = 30	0.96 (0.92 to 0.98)	0.76	SPADI	-	-
	(20-80)					*r* = –0.80		
						p < 0.001		
Padua R et al. 2010	n = 50	Italian	7 days n = 20	0.91	0.85	DASH		
	(33-78)					*r* = –0.92	*r = 0.48*	*r* = –0.20
						p < 0.02	*p < 0.01*	ns
						OSQ		
						*r* = 0.78		
						p < 0.02		
Goldhahn J et al. [7]	n = 118	German	7 days	0.93 (0.90 to 0.95)	0.96	SPADI		
						*r* = 0.92	*r* = 0.64	Overall SF-36
	(33 to 89)							
						DASH		*r* = 0.66
						*r* = 0.84		
Kocher et al. [9]	n = 1066	Validation study English	4 weeks	0.94 (n = 56) age range 15-78 years	0.61 instability 0.64 rotator cuff disease 0.62 arthritis	*-*	SF-12	SF-12
							*r* = 0.32-0.58	*r* = –0.09-0.11
	(13-95)							
							p < 0.001-0.002	p = 0.27-0.67
Michener et al. [10]	n = 63	Validation study English	24 to 72 hours, and after 3 to 4 weeks	0.84 (0.75 to 0.91)	0.86	Penn Score		
	(20-81)							
						*r* = 0.78	*r* = 0.40	*r* = 0.15
						p < 0.01	p = 0.001	p = 0.25

*ASES* American Shoulder and Elbow Surgeons Standardized Shoulder Assessment Form, *ICC* intra-class correlation coefficient, *Cl* Confidence interval, SST, Simple Shoulder Test, *SPADI* Shoulder Pain and Disability Index, *DASH* Disability of Arm, Shoulder and Hand questionnaire, *OSQ* Oxford Shoulder Questionnaire, *Penn Score* the University of Pennsylvania Shoulder Score, *SF-36* Short Form 36 Health Survey, *PCS* Physical Component Summary, *MCS* Mental Component Summary, *SF-12* Short Form 12 Health Survey.

In the present study, the internal consistency of the ASES was good, which indicates that several items that propose to measure the same general construct produce similar scores. The α-values measuring internal consistency varied considerably ranging from 0.61 to 0.96 in the previous studies [6, 7, 9, 10, 12] demonstrating that the homogeneity of the ASES items in a scale varies in all the studies (Table 4). The main reason for this may be the differences in the study samples. The recommended Cronbach’s alpha for group comparisons is higher than 0.80 [20]. However, “very good” internal consistency may indicate that the items are too homogenous. From that point of view, our study expresses good reliability and demonstrates that the items of the Finnish ASES are reasonably related and still contribute unique information about the patient’s status. In the present study, the factor analysis showed unidimensionality of the ASES. However, it has been suggested that factor analysis for the ASES was loaded in 2 dimensions [12]. The reason for this may be due to study group differences.

Our a priori hypotheses were accomplished, as the ASES questionnaire had a strong correlation with the SST, the Physical Component Score of the SF-36, and also with the single disability question (expressed on a visual analogue scale). This confirmed the construct validity and reassured us that these measurement procedures were measuring the same construct. In the previous studies, correlations between the ASES and other shoulder-specific or upper limb-specific questionnaires have been strong [6, 7, 10, 12]. Correlation between the SST and the ASES has been found to be strong, which is consistent with the similarity in their constructs [21]. In the present study the SST score was more related to function score than pain score of the ASES (Table 3). The reason for this may be the fact that a half of the ASES consists of single value of pain VAS and another half consists of function score that is quite similar to the SST. There was not a statistically significant correlation between the ASES questionnaire and the Mental Component Score of the SF-36 (Table 3). This result demonstrates that the ASES disability questionnaire and the Mental Component Score of the SF-36 questionnaire do not measure the same entity. On the contrary, Çelik et al. [6] reported significant correlation between the ASES and the Mental Component Score of the SF-36, meanwhile correlation between the ASES and the Physical Component Score of the SF-36 was weak (Table 4). The differences in correlations may be due to differences in, e.g. sample size, age, reason for shoulder disorder.

The questionnaire showed to be highly acceptable, easily understood, and capable of being self-administered. Any suggestions for improving the wording were not given, except the question about lifting 10 lbs above the shoulder was adapted to the metric system. Thus, the weight is 4 kg in our study. A variance of 4 to 5 kg has been used in most of the studies concerning the validation of the ASES questionnaire [6–9, 11].

The strength of the present study is that the subjects represented a very large range of ages and many different shoulder diagnoses. Another strength of this study is that the patients were grouped into stable, improved, and worsened categories. Using this subgroup analysis, we could assess the patients whose symptoms had changed. Furthermore, earlier literature has recommended that functional status questionnaires be measured within a 2-week time interval to test their reproducibility [14]. In our study, the patients completed the ASES questionnaire twice: 2 weeks before and at the time of their arrival to the outpatient clinics of physical medicine and rehabilitation or orthopaedics and traumatology. This procedure was applied to minimise the possibility that the patients received new treatments, which would potentially influence the responses of the second assessment, between these two time points.

A limitation of our study is that it was performed in a hospital setting. The patients were collected from the outpatient clinics of a single hospital following referral to specialised care. The patients had chronic shoulder problems, and they were examined by specialists. Thus, the sample assessed in this study may not represent subjects with shoulder pain in the entire population.

## Conclusions

The self-report section of the Finnish ASES is a reliable and valid tool and can therefore be used as an instrument to assess shoulder disability among Finnish patients of different ages with different shoulder diagnoses.

## Electronic supplementary material

Additional file 1: **ASES suomi that presents the Finnish American Shoulder and Elbow Surgeons Standardized Shoulder Assessment Form.** (DOCX 122 KB)

## Abbreviations

ASES: American Shoulder and Elbow Surgeons Standardized Shoulder Assessment Form
SST: Simple Shoulder Test
SF-36: Short-Form 36 Health Survey
EuroQol: European Quality of life scale
WHOQOL: World Health Organization Quality of Life
HRQOL: Health-Related Quality of Life.
