# Supplementary material for: Reliability and validity of the Finnish version of the American Shoulder and Elbow Surgeons Standardized Shoulder Assessment Form, patient self-report section
Source: BMC Musculoskelet Disord. 2014 Aug 11;15:272. doi: 10.1186/1471-2474-15-272 (PMC4243510; doi:10.1186/1471-2474-15-272)
Supplement: Supplementary file 1 — Additional file 1: ASES suomi that presents the Finnish American Shoulder and Elbow Surgeons Standardized Shoulder Assessment Form. (DOCX 122 KB) [file 12891_2014_2220_MOESM1_ESM.docx]

| ASES suomi | | | | | | |
| --- | --- | --- | --- | --- | --- | --- |
| **KYSELYLOMAKE OLKANIVELPOTILAALLE** | | | | | | |
| Onko sinulla tällä hetkellä olkakipua? (ympyröi oikea vastaus) | | | | Kyllä | | Ei |
| Merkitse viereiseen kuvaan kivulias alue: | |  | | | | |
| Onko sinulla olkakipua öisin? | | | | Kyllä | | Ei |
| Käytätkö tavallisia kipulääkkeitä (Burana, Ibumax, Voltaren, Ketorin, Paratabs)? | | | | Kyllä | | Ei |
| Käytätkö vahvoja kipulääkkeitä olkakivun takia (Tramal, Panacod jne.)? | | | | Kyllä | | Ei |
| Montako kipulääketablettia otat keskimäärin / päivä | | | | tablettia | | |
| Olkakipusi tänään (merkitse kiputasosi janalle poikkiviivalla)?  0 10  Ei kipua Pahin mahdollinen kipu | | | | | | |
| Tuntuuko olkanivelesi löysältä? (tuntuu kuin nivel menisi sijoiltaan) | | | | Kyllä | | Ei |
| Kuinka löysältä olkanivelesi tuntuu? (merkitse janalle poikkiviivalla)  0 10  Erittäin tukeva Erittäin löysä | | | | | | |
| Seuraavilla kysymyksillä selvitetään olkanivelesi toimintaa tällä hetkellä (ympyröi sopiva vaihtoehto): 0 **=** **En pysty**; **1 =** **Pystyn, mutta paljon vaikeuksia**  **2 =** **Hieman vaikeuksia**; **3 =** **Ei ongelmia (normaali tilanne)** | | | | | | |
| **PYSTYTKÖ** | | | **Oikea yläraaja** | | **Vasen yläraaja** | |
| 1. pukemaan takin (käden pujottaminen hihaan)? | | | 0 1 2 3 | | 0 1 2 3 | |
| 2. nukkumaan kyljellä, jos kipeä olkapää on alla? | | | 0 1 2 3 | | 0 1 2 3 | |
| 3. viemään kätesi alakautta selän taakse lapojen väliin? | | | 0 1 2 3 | | 0 1 2 3 | |
| 4. huolehtimaan WC-käynneillä henkilökohtaisesta hygieniasta (pyyhkiminen ulostamisen jälkeen)? | | | 0 1 2 3 | | 0 1 2 3 | |
| 5. kampaamaan hiuksesi vieden käden pään päälle? | | | 0 1 2 3 | | 0 1 2 3 | |
| 6. kurkottamaan tavaroita korkealta hyllyltä? | | | 0 1 2 3 | | 0 1 2 3 | |
| 7. nostamaan 4 kg esineen olkapäätason yläpuolelle? | | | 0 1 2 3 | | 0 1 2 3 | |
| 8. heittämään palloa yläkautta? | | | 0 1 2 3 | | 0 1 2 3 | |
| 9. suoriutumaan normaaleista töistäsi (normaalit työt = ammatti ja kotityöt ennen olkavaivan alkamista)? | | | 0 1 2 3 | | 0 1 2 3 | |
| 10. harrastamaan normaalia liikuntaasi (normaali liikunta = liikunta ennen olkavaivan alkamista)? | | | 0 1 2 3 | | 0 1 2 3 | |
